# Single cell transcriptomics reveal trans-differentiation of pancreatic beta cells following inactivation of the TFIID subunit Taf4

**DOI:** 10.1038/s41419-021-04067-y

**Published:** 2021-08-12

**Authors:** Thomas Kleiber, Guillaume Davidson, Gabrielle Mengus, Igor Martianov, Irwin Davidson

**Affiliations:** 1 Institut de Génétique et de Biologie Moléculaire et Cellulaire. BP 163, 67404 Illkirch Cedex, C.U Strasbourg, France; 2 Centre National de la Recherche Scientifique, UMR7104 Illkirch, France; 3 Institut National de la Santé et de la Recherche Médicale, U1258 Illkirch, France; 4 Université de Strasbourg, Illkirch, France; 5 Orphazyme, Ole Malloes Vej 3, 2200 Copenhagen, Danmark; 6 Equipe Labélisée Ligue National contre le Cancer, Alsace, France

**Keywords:** Transcriptomics, Endocrinology

## Abstract

Regulation of gene expression involves a complex and dynamic dialogue between transcription factors, chromatin remodelling and modification complexes and the basal transcription machinery. To address the function of the Taf4 subunit of general transcription factor TFIID in the regulation of insulin signalling, it was inactivated in adult murine pancreatic beta cells. Taf4 inactivation impacted the expression of critical genes involved in beta-cell function leading to increased glycaemia, lowered plasma insulin levels and defective glucose-stimulated insulin secretion. One week after Taf4-loss, single-cell RNA-seq revealed cells with mixed beta cell, alpha and/or delta cell identities as well as a beta cell population trans-differentiating into alpha-like cells. Computational analysis of single-cell RNA-seq defines how known critical beta cell and alpha cell determinants may act in combination with additional transcription factors and the NuRF chromatin remodelling complex to promote beta cell trans-differentiation.

## Introduction

Pancreatic Langerhans islets comprise multiple hormone-secreting cell types that cooperate to regulate glucose homeostasis in the organism. Plasticity and trans-differentiation between endocrine cell populations have been described in diabetes, genetically modified mice and after various pathological or chemical treatments [1–6]. Alpha cells have a plastic epigenetic state with many loci showing bivalent chromatin modifications [7] and can be converted to beta-like insulin-secreting cells by overexpression of beta cell-determining factors [8] or loss of Arx and Dnmt1 [9]. In type 2 diabetes, hyperglycaemia may induce beta-cell dedifferentiation through reversion to a Ngn3-expressing precursor state [1, 10–13]. However, with some exceptions [9, 14, 15], the transcriptional mechanisms underlying changes in cell identity are not fully understood.

Regulation of gene expression requires a dynamic dialogue between transcription factors binding cis-regulatory elements, chromatin remodelling and modification complexes and the basal transcription machinery [16–20]. Basal transcription factor TFIID comprising the TATA-box binding protein (TBP) and 13–14 TBP-associated factors (TAFs) plays a critical role in this communication and in pre-initiation complex (PIC) formation [21, 22]. TFIID is organised in three lobes A–C [22–24]. The histone-like TAF4-TAF12 heterodimer is crucial for the structural integrity of lobes A and B [25, 26]. In lobe B, the conserved TAF4 C-terminal domain contacts promoter DNA and the TFIIA-TBP module suggesting they promote TBP DNA binding and fix the distance between the TBP binding and the transcription start sites.

Genetic Taf4 inactivation in mouse leads to specific effects on gene expression that may in part be explained by redundancy with its paralog TAF4B that maintains TFIID integrity in absence of TAF4 [27, 28]. We used somatic inactivation to address Taf4 function in the embryonic and adult epidermis or neonatal hepatocytes [26, 27, 29–31] and germline inactivation to address its role during embryogenesis [28]. In each biological context, Taf4 regulated specific gene expression programs and functions, in particular in advanced stages of embryonic tissue differentiation.

We used Insulin-Cre-ER^T2^ transgenics to inactivate Taf4 in murine adult beta cells showing that it regulates an extensive gene expression program including critical components of the insulin signalling pathway. Taf4 loss also impacts beta cell identity and computational analysis of single-cell transcriptomics data defined how known beta and alpha cell determinants may act in combination with additional transcription factors and the NuRF chromatin remodelling complex to promote beta-cell trans-differentiation into alpha-like glucagon-expressing cells.

## Materials and methods

### Genetically engineered mice

Mice with the floxed Taf4 alleles as previously described [27] were crossed with previously described [32] *Ins*-Cre-ER^T2^ transgenics and Taf4 inactivated by subcutaneous injection 1 time per day for 3 days with 5 μl of Tamoxifen diluted in oil (final concentration 10 mg/ml). Fasting blood glucose and insulin levels were measured over a period of 20 weeks with one measurement per week on the same day at the same time. Glycaemia was measured using a glucometer and insulin levels using the Milliplex Map Kit (Mouse Metabolic Magnetic Bead Panel. MMHMAG-44K, Millipore). To assess glucose tolerance, mice were fasted for 12 hours before the first glucose measure and then injection of 100 μl of glucose solution (t= 0, 2 g of glucose/ kg mouse weight). Insulin levels were monitored in blood samples taken over a 30-min period starting 5 min before the glucose injection. The analysed cohort comprised animals from 6 litters generated by independent sets of Tam injections over a period of more than 1 year. All animals were at the same age at the time of Tam injection. Littermates of these animals were sacrificed for the histology and RNA-seq experiments. In total, more than 7 litters (more than 60 individual mice) from independent crosses were used in this study. Animals with the correct genotypes were randomly chosen from each litter for all types of experiments. No animals were excluded from experimentation unless they were evidently in poor health.

All animal experiments were performed in accordance with European and national guidelines and policies (2010/63/UE directive and French decree 2013–118) and with approval of the National Ethics Committee.

### Immunostaining

Pancreas were dissected and fixed overnight in 4% paraformaldehyde, washed with PBS, dehydrated, paraffin embedded, and sectioned at 5 μm. For antigen retrieval, the sections were boiled for 20 min in 10 mM of sodium citrate buffer. Sections were permeabilized with 3 × 5 min 0.1% Triton in PBS, blocked for 1 h in 5% Neutral Goat Serum (NGS) in PBS, and incubated overnight in 5% NGS with primary antibodies: mouse anti-Glucagon [K79bB10] (Abcam, ab10988), Guinea Pig anti-Insulin (Dako IR00261-2) and mouse anti-TAF4 (TAF II p135) (Santa Cruz (sc-136093). Sections were washed 3 × 5 min 0.1% Triton in PBS, and incubated with secondary antibodies: Goat anti-Guinea pig IgG H&L Alexa Fluor® 647 (Abcam ab150187) and Goat anti-Mouse IgG H&L Alexa Fluor® 488 (Abcam ab150117) for 2 h. Sections were subsequently incubated with 1/2000 Hoechst nuclear stain for 10 min, washed 3 × 5 min in PBS, dried, and mounted with Vectashild. For Taf4 and Gcg co-staining the guinea pig anti-Gcg antibody (LINCO, 4031-01 F) was used after 20 min of epitope retrieval. For GFP and Gcg co-staining, frozen cryostat sections were used without epitope retrieval. Gcg was detected with mouse anti-Glucagon [K79bB10] (Abcam ab10988) and chicken anti-mouse IgG Alexa Fluor® 647 (Thermo Scientific, # A-21463).

### Isolation of intraductal Langerhans islets

Anesthetized mice (40 μL ketamine (100 mg/mL) + 24 μL xylazine (20 mg/mL) + 160 μL NaCl intraperitoneal injection per mouse) were injected in the pancreas with 4 ml of collagenase solution (7 ml HBSS, 70 μL HEPES (10 μM) (Gibco), 16,5 μL DNAse (10 mg/mL) (sigma DN5), 14 mg collagenase (2 mg/mL) (sigma Type V), and for isolation of RNA 2.3 μL of RNAsine (40U/μL). The pancreas was dissected and placed a tube containing 2 ml collagenase solution and incubated at 37 min at 38 °C before stopping with 30 mL Quenching Buffer (QB; 122 mL HBSS + 2.9 mL HEPES (25 μM) + 0.6 g BSA (0.5%) and 2 cycles of centrifugation at 1200 rpm for 2 min at 4 °C. The pellet was suspended in 10 ml of Histopaque 1100 (5 ml histopaque 1077; Sigma 10771 and 6 ml histopaque 1119; Sigma 11191), centrifuged for 20 min at 1500 rpm at 4 °C and the pellet washed two times with QB. The supernatant was discarded and pelleted islets transferred to a bacterial culture dish to be collected by pipetting. Purified islets were then cultured for 24 h in RPMI1640 (HEPES 25 mM) + final 2 mM L-glutamine (Gibco 11875-093) + 10% FCS 95 + 1% Penicillin/Streptomycin. Intracellular ATP concentration was measured with the Luminescent ATP Detection Assay Kit (ab113849) on purified islets that were further dissociated to obtain a single-cell population and sorted by flow cytometry to normalize the numbers of cells used on a LSRII Fortessa (BD Biosciences).

### Bulk RNA-seq from isolated islets

RNA was extracted from islets from 2 mice per genotype for each biological replicate with Nucleospin RNA plus XS (Macherey-Nagel). Complementary DNA was generated and linearly amplified from 3 ng total RNA using the Ovation RNA-seq V2 system (NuGEN technologies Inc., Leek, The Netherlands), according to the manufacturer’s instructions. The amplified cDNA was then purified using Agencourt AMPure XP beads (Beckman-Coulter, Villepinte, France) in a 1.8:1 bead to sample ratio and fragmented by sonication using a Covaris E220 instrument (with duty cycle: 10%, maximum incident power: 175 watts and cycles/burst: 200 for 120 s). The RNA-seq libraries were generated from 100 ng fragmented cDNA using the Ovation Ultralow v2 library system (NuGEN technologies Inc., Leek, The Netherlands) according to the manufacturer’s instructions, with only 6 PCR cycles for library amplification. The final libraries were verified for quality and quantified using capillary electrophoresis before sequencing on an Illumina Hi-Seq4000. and Reads were preprocessed using cutadapt version 1.10 in order to remove adapter and low-quality sequences (Phred quality score below 20). After this preprocessing, reads shorter than 40 bases were discarded. Reads were mapped to rRNA sequences using bowtie version 2.2.8, and reads mapping to rRNA sequences were removed. Reads were mapped onto the mm9 assembly of Mus musculus genome using STAR version 2.5.3a. Gene expression quantification was performed from uniquely aligned reads using htseq-count version 0.6.1p1, with annotations from Ensembl version 67 and “union” mode. Only non-ambiguously assigned reads were retained. Read counts were normalized across samples with the median-of-ratios method [33]. Comparisons of interest were performed using the Wald test for differential expression [34] and implemented in the Bioconductor package DESeq2 version 1.16.1. Genes with high Cook’s distance were filtered out and independent filtering based on the mean of normalized counts was performed. *P*-values were adjusted for multiple testing using the Benjamini and Hochberg method [3]. Heatmaps were generated with R-package pheatmap v1.0.12. Deregulated genes were defined as genes with log2(fold change) >1 or <−1 and adjusted *p*-value < 0.05. Gene ontology analyses were performed using GSEA (https://www.gsea-msigdb.org/gsea/index.jsp) or David (https://david-d.ncifcrf.gov/).

### ATAC-seq from isolated islets

ATAC-seq was performed from 20,000 cells from isolated islets. Sequenced reads were mapped to the mouse genome assembly mm9 using Bowtie [35] with the following arguments: “-m 1 --strata --best -y -S -l 40 -p 2”.

After sequencing, peak detection was performed using the Malpha cellS software [36] v2.1.1.20160309. Peaks were annotated with Homer(http://homer.salk.edu/homer/ngs/annotation.html) using the GTF from ENSEMBL v67. Peak intersections were computed using Bedtools [37] Global Clustering was done using seqMINER [38]. In silico footprinting signatures were calculated using TOBIAS [39] (v0.5.1; https://github.com/loosolab/TOBIAS/), differential footprinting scores were plotted with R-package ggplot2 (https://ggplot2.tidyverse.org.).

### Single-cell RNA-seq from dissociated islets

After islet dissociation, further dissociation was performed with Accutase (A6964, Sigma) at 27 °C for 5 min and the cells were suspended in culture medium. Cells were then sorted by flow cytometry to select only live cells. Three hundred islets from a male WT mouse and 300 islets pooled from two male mutant mice 1 week or 5 weeks after Taf4 inactivation were used for scRNA-seq. Cell capture was performed using 10X Genomics Chromium Analyzer. After sequencing, raw reads were processed using CellRanger (v 3.1) to align on the mm10 mouse genome, remove unexpressed genes and quantify barcodes and UMIs. Data were then analysed in R (v3.6.3). For the WT sample only, potential doublets were removed by manually filtering out cells expressing high levels of Ins1 + Gcg, Ins1 + Sst or Gcg + Sst and then by running R-package DoubletFinder [40]. The WT sample was down-scaled to 12,000 cells before aggregation with Taf4^b−/−^ 5-week sample and analysed using Seurat v3.1.4 [41] following the guided clustering tutorial vignette. Samples were first aggregated with the “CellRanger aggr” and then normalized and scaled together in Seurat. Only cells with feature count ranging from 200 to 1500 and with percentage of mitochondrial reads <15% were kept for the analysis. Counts are normalized with the “LogNormalize” method and data are scaled to remove unwanted sources of variation. Clustering was done on most variable features using 12 PCs and a resolution of 0.8. Regulome analyses were performed using the SCENIC v1.1.2.2 package.

## Results

### Inactivation of Taf4 in adult pancreatic beta cells

**Fig. 1:**
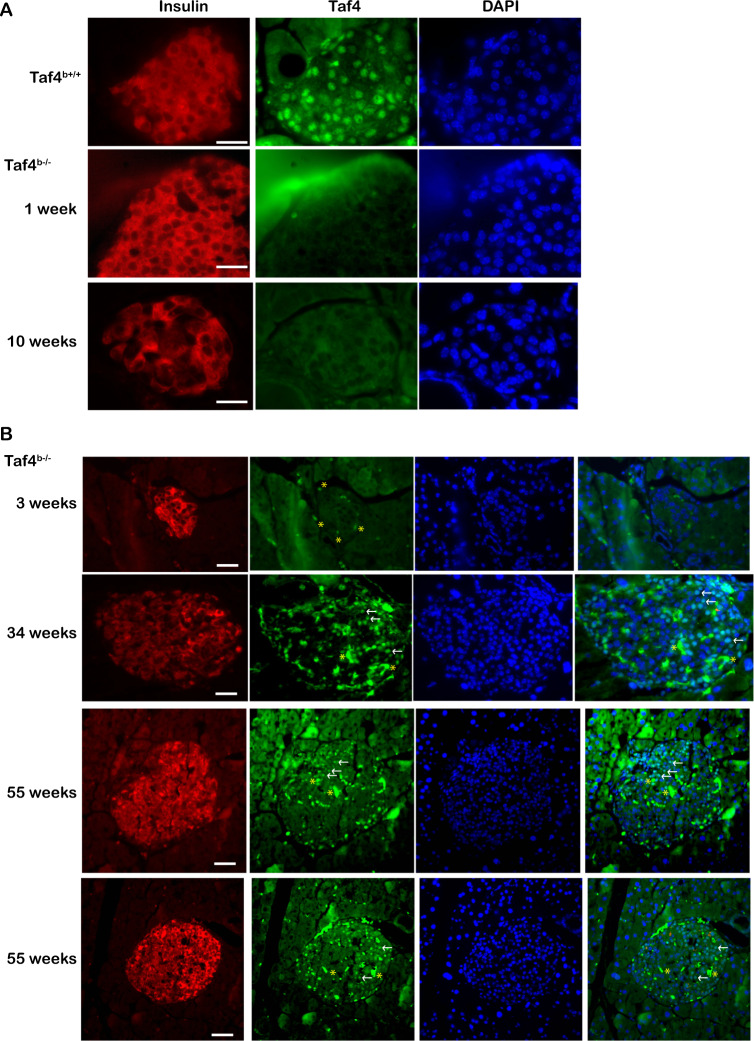
Inactivation of Taf4 in beta cells. **A** Immunostaining of Langerhans islets from mice with the indicated genotypes for Taf4 or insulin as indicated. The number of weeks after Tam injection are indicated. **B** Immunostaining of Langerhans islets as above but with the addition of the Taf4-DAPI merge. Representative Taf4-positive nuclei at 34 and 55 weeks are indicated by arrows, not to be confounded with the stronger non-specific staining indicated by *. Scale bar = 100 μM.

To inactivate Taf4 selectively in adult pancreatic beta cells, mice with floxed Taf4 alleles [26, 27] were crossed with an *Ins1*::Cre-ER^T2^ transgenic driver [32] to generate *Ins1*::Cre-ER^T2^::*Taf4*^lox/lox^ or *Taf4*^+/+^ animals. At 11–13 weeks of age, animals of each genotype were injected with Tamoxifen (Tam) on 3 consecutive days to generate *Taf4*^b−/−^ mice lacking Taf4 in beta cells and control *Taf4*^+/+^ animals maintaining Taf4 expression. Islet immunostaining revealed Taf4 loss 1 week after injection in *Taf4*^b−/−^ animals, whereas it was clearly visible in Tam injected *Ins1*::Cre-ER^T2^::*Taf4*^+/+^ controls (Fig. 1A). No Taf4 was detected in *Taf4*^b-/-^ animals 3 and 10 weeks after Tam injection, but it reappeared in a sub-population of beta cells after 34 weeks (Fig. 1A and B and Supplementary Fig. 1A). After 55 weeks, islets comprised Taf4-expressing and non-expressing beta cells with insulin staining being more intense in Taf4-expressing beta cells.

Loss of Taf4 in all islet cells was inconsistent with the use of the *Ins1*::Cre-ER^T2^ transgenic driver as its expression should persist in alpha cells. As antigen retrieval treatment necessary for Taf4 staining may result in loss of peripheral alpha cells, we used frozen islet sections. Upon Taf4 and Gcg co-staining, nuclear Taf4 was seen in *Taf4*^+/+^ beta cells and co-staining with Gcg in peripheral alpha cells (Supplementary Fig. 1B). Taf4 expression persisted in *Taf4*^b−/−^ Gcg-expressing cells, but was lost from the beta cells showing selective Taf4 inactivation in beta cells.

### Defective glucose-stimulated insulin secretion upon Taf4 inactivation

**Fig. 2:**
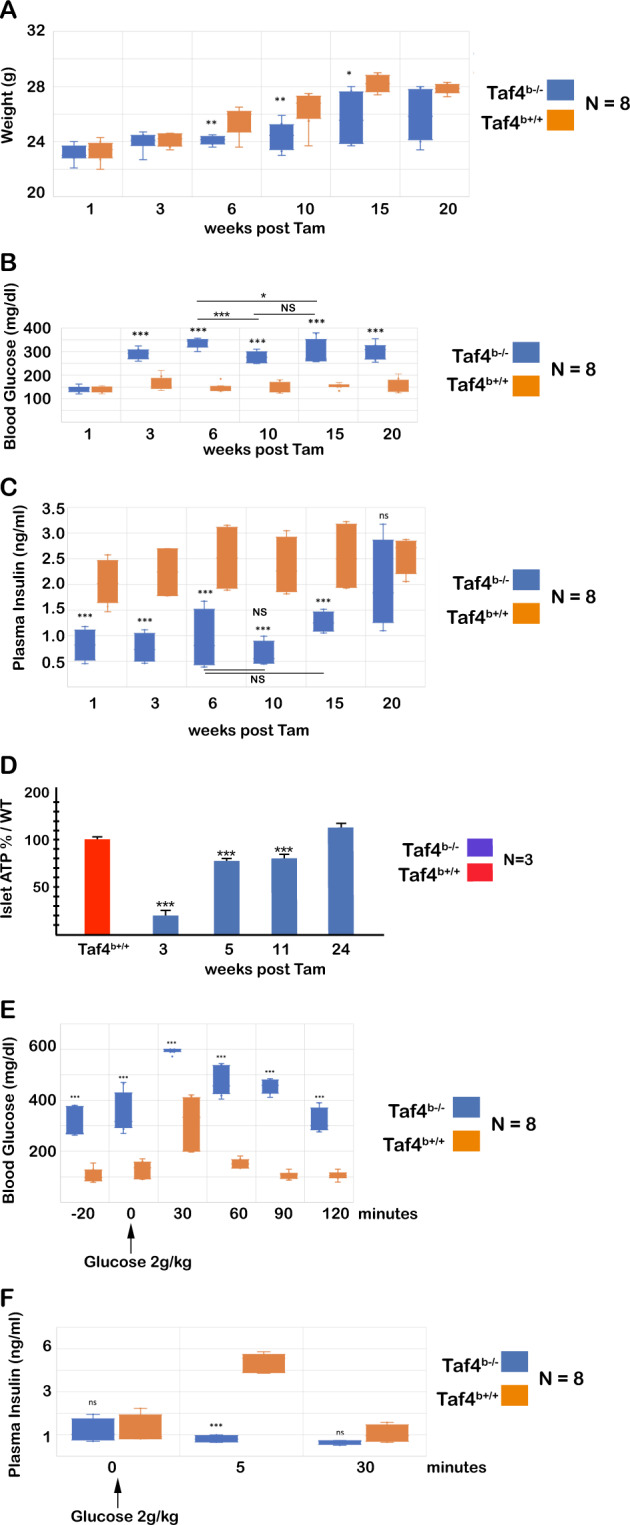
Physiological parameters of *Taf4*^*b−/−*^ animals. **A** Weight of *Taf4*^*b*+/+^ and *Taf4*^*b−/−*^ animals at the indicated number of weeks following Tam injection. **B**, **C** Blood glucose and insulin levels of *Taf4*^*b*+/+^ and *Taf4*^*b−/−*^ animals at the indicated number of weeks following Tam injection. The * above the bars indicate the significant difference between *Taf4*^*b*+/+^ and *Taf4*^*b−/−*^ animals at each time point, whereas * and NS above/below the lines indicate significant or non-significant differences between the *Taf4*^*b−/−*^ values at the indicated times. **D** ATP content of isolated islets from control animals or from *Taf4*^*b−/−*^ animals at the indicated number of weeks following Tam injection. Values are expressed as a % of the *Taf4*^*b*+/+^ values at each time point taken as 100%. **E** Blood glucose levels at the indicated times following injection of glucose in control and *Taf4*^*b−/−*^ animals. Mice at 14 weeks were injected with Tam and glucose administered 3 weeks following Tam treatment. **F** Plasma insulin levels from the same protocol. *T*-test with two-tailed *P*-value analyses and confidence interval 95% were performed by Prism 5. *P*-values: *p < 0.05; **p < 0.01; ***p < 0.001. Data are mean ± SD from *N* = 8 or *N* = 3 as indicated.

We examined different physiological parameters over 20 weeks following Taf4 inactivation. Compared to *Taf4*^+/+^ littermates, *Taf4*^b−/−^ animals showed progressive reduction in body weight (Fig. 2A). Fasting blood glucose levels in otherwise ad libitum-fed mice indicated a potent increase in glycaemia after 3 weeks (Fig. 2B) that persisted over 20 weeks, although levels were mildly but significantly reduced after 10 weeks. Plasma insulin levels were also reduced between weeks 1–10, but recovered to those of *Taf4*^+/+^ animals at later times (Fig. 2C). Islet ATP levels strongly decreased after 3 weeks in the *Taf4*^b−/−^ animals, followed by progressive recovery between 11 and 24 weeks (Fig. 2D). Loss of Taf4 therefore resulted in increased glycaemia and lowered plasma insulin that were partially restored after 10 weeks. As a result, *Taf4*^b−/−^ animals did not show total loss of beta-cell function and no premature death was seen with only rare Taf4-expressing or mutant mice dying over 14 months of surveillance.

Taf4 loss further led to defective glucose-stimulated insulin secretion (GSIS). Fasting *Taf4*^b−/−^ animals and their *Taf4*^+/+^ littermates 3 weeks after Tam injection were injected with glucose and the resulting plasma glycaemia and insulin levels were monitored. Before injection, the *Taf4*^b−/−^ animals showed elevated glycaemia that was further increased 30 min after glucose administration (Fig. 2E) and persisted over 90 min returning to basal values by 120 min. In contrast, their *Taf4*^+/+^ littermates showed a lower increase in glycaemia that returned to basal values after 90 min. Analogous results were seen using animals 2 and 4 weeks after Tam injection (data not shown). Similarly, glucose administration in *Taf4*^b−/−^ animals failed to increase plasma insulin levels as seen in *Taf4*^+/+^ animals (Fig. 2F).

### Taf4 regulated gene expression programs in beta cells

**Fig. 3:**
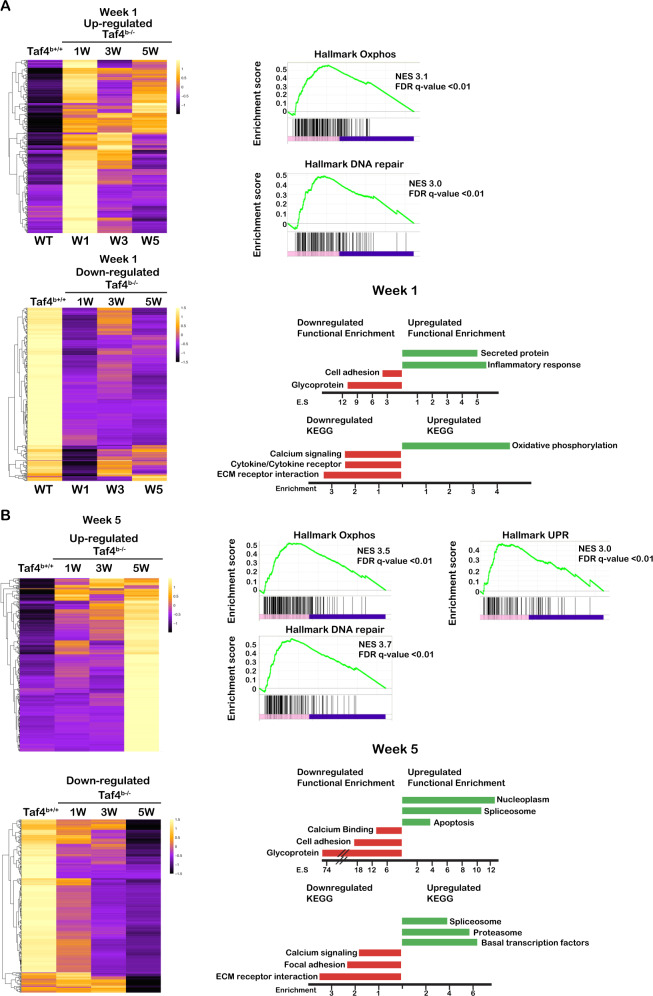
Effects of Taf4 inactivation on islet gene expression. **A** Heatmap showing the expression of the 100 genes most up and downregulated 1 week after Taf4 inactivation. Right hand panels show the GSEA and ontology analyses of the de-regulated genes. **B** Heatmap showing the expression of the 100 genes most up and downregulated 5 weeks after Taf4 inactivation. Right hand panels show the GSEA and ontology analyses of the de-regulated genes.

We performed RNA-seq from *Taf4*^+/+^ islets and from *Taf4*^b−/−^ islets 1, 3 and 5 weeks after Tam injection (Fig. 3A, B and Supplementary Dataset 1). At 1 week, around 1000 genes were up-or down-regulated (Fig. 3A and Supplementary 2A, B). At 3 and 5 weeks, the number of de-regulated genes strongly increased (Fig. 3A and Supplementary 2A, B). More than 300 genes were downregulated under all conditions. At 1 week, genes critical for beta-cell function such as *Slc2a2*, *Trpm5*, *Ins1* or involved in calcium signalling and cell contact were downregulated (Supplementary Fig. 2C). Upregulated genes were strongly enriched in inflammatory/stress response including numerous chemokines and *Reg3a*, *Reg3b* and *Reg3g* (Supplementary Fig. 2C) as well as in oxidative phosphorylation, DNA metabolism and general transcription factors (Fig. 3A and Supplementary Dataset 1). Expression of the alpha and delta cell markers *Gcg* and *Sst* was also rapidly upregulated and then attenuated with time (Supplementary Fig. 2C).

While an increasing number of genes was deregulated over time, we identified sets of genes whose normal expression was partially restored. Expression of a subset of genes strongly downregulated after 1 week was progressively restored after 3 and 5 weeks (Fig. 3A and Supplementary 2D). Similar results were seen with upregulated genes the most striking example being the inflammatory/stress response genes upregulated after 1 week but restored to almost normal after 3 and 5 weeks.

Upregulation of genes involved in chromatin organisation, RNA metabolic functions and transcription were observed after 3 weeks and persisted at 5 weeks (Fig. 3B and Supplementary 2D), suggesting that the acute stress response was followed by chromatin reorganisation and compensation for loss of *Taf4* by upregulation of *Taf4b* and *Taf12*. Indeed, *Taf4b* expression was much lower than that of *Taf4* in wild-type islets.

ATAC-seq on isolated islets revealed a global increase in accessibility after 1 week, but at the proximal promoter, the number of accessible sites was rather enriched in *Taf4*^+/+^. (Supplementary Fig. 3A). A similar result was seen after 5 weeks (Supplementary Fig. 3B). To identify transcription factors showing differential occupancy, we carried out in silico footprinting analyses at the proximal promoter [39]. Binding of Hox-domain containing transcription factors including critical beta-cell factors Nkx6-1 and Hnf1b was enriched in *Taf4*^+/+^, whereas Yy2, Arnt2, Bhlhe41 and Mycn were enriched in *Taf4*^−/−^ (Supplementary Fig. 3C). Similarly, after 5 weeks, enriched binding of several factors including Yy2, Nrf1 was observed (Supplementary Fig. 3D).

ATAC-seq and RNA-seq data indicated that Taf4 loss led to reduced chromatin accessibility at proximal promoters with reduced binding of critical beta-cell factors together with expression of genes essential for beta-cell function, but enhanced binding of a new set of transcription factors.

### Taf4 inactivation results in loss of beta-cell identity and trans-differentiation into alpha-like cells

While the above data provided an overall view of gene expression changes in islets, they did not assess whether Taf4 loss affected only beta-cell gene expression or if it impacted gene expression in other cell types as suggested by increased *Gcg* and *Sst* expression. To address this, we performed single-cell (sc)RNA-seq from purified dissociated Langerhans islets from *Taf4*^+/+^ and *Taf4*^*b−/−*^ mice 1 and 5 weeks after Tam injection.

**Fig. 4:**
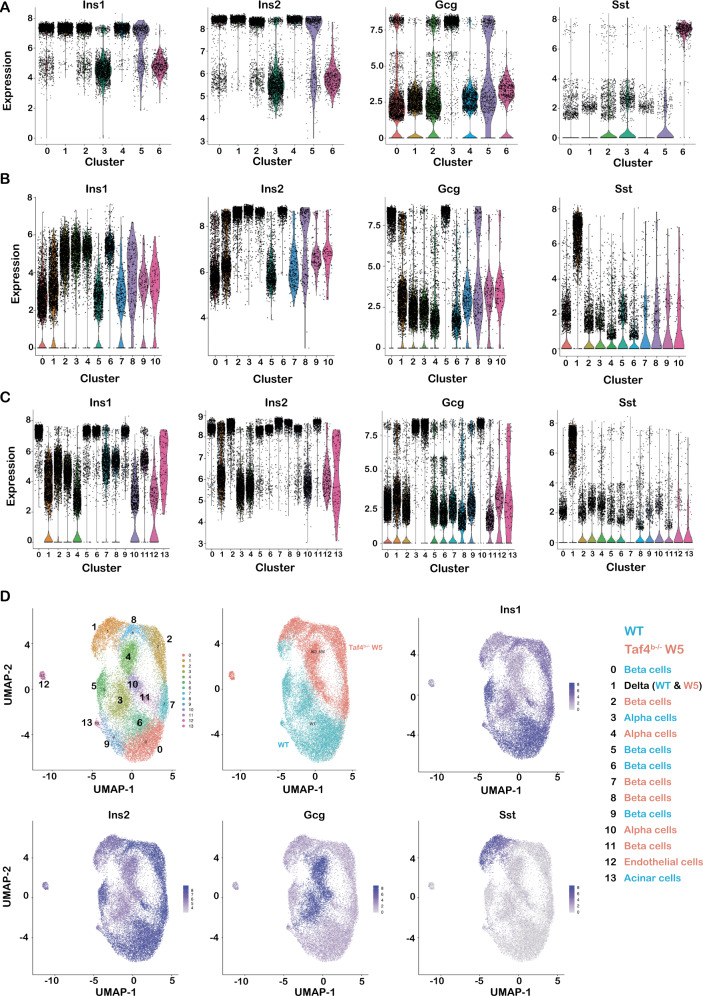
Sc-RNA-seq of WT and week 5 mutant islets. **A**–**C** Violin plots of expression of the indicated genes in each cluster from WT islets (**A**), 5-week mutant islets (**B**) and the aggregate (**C**). **D** UMAP representations of the aggregate data, illustrating origin of cells from WT and mutant animals and the expression of the genes indicated in each panel.

Analyses of around 12,000 cells from *Taf4*^+/+^ islets identified the major endocrine populations with 4 beta cell clusters expressing high *Ins1* and *Ins2* (clusters 0–2 and 4 in Fig. 4A). Cluster 3 represented *Gcg*-expressing alpha cells, cluster 6 *Sst*-expressing delta cells and cluster 5 an acinar population (Fig. 4A). The 4 beta-cell sub-populations were distinguished by a strong ER stress signature in cluster 2, while cluster 0 represented potential secretory cells with high *Slc2a2*, *Abcc8* and *G6pc2* and lower *Ins1*/*2* that was higher in clusters 1 and 4 (Supplementary Fig. 4).

We analysed 10,666 cells 5 weeks after Taf4 inactivation distinguishing 4 beta cell populations with high *Ins2* (clusters 2–4 and 6, Fig. 4B), but reduced *Ins1* as noted in the bulk RNA-seq. Cluster 1 corresponded to *Sst*-expressing delta cells, while clusters 0 and 5 corresponded to *Gcg*-expressing alpha cells. Aggregation of *Taf4*^+/+^ and week 5 data sets (Fig. 4C, D and Supplementary Fig. 5) clearly distinguished *Taf4*^+/+^ beta cell populations from *Taf4*^−/−^ based on high *Ins1* expression in *Taf4*^+/+^ (clusters 0, 5, 6 and 9) from *Ins2* high/*Ins1* low *Taf4*^−/−^ (clusters 2, 7, 8 and 11). The cell populations segregated in a UMAP representation (Fig. 4D) where *Taf4*^+/+^ and *Taf4*^−/−^ beta cells were further distinguished by strongly reduced *Ucn3* and *Slc2a2* expression (Supplementary Fig. 6A). High expression of stress markers *Ddit3* and *Herpud1* characterized *Taf4*^+/+^ beta cells in cluster 5 and *Taf4*^−/−^ clusters 2 and 8 (Supplementary Fig. 6A). The aggregate also revealed 3 distinct alpha cell populations, cluster 3 from *Taf4*^+/+^ and clusters 4 and 10 from *Taf4*^−/−^ with a strong stress response in cluster 4 (Supplementary Fig. 5). Delta cells from the two conditions were more homogenous (cluster 3).

**Fig. 5:**
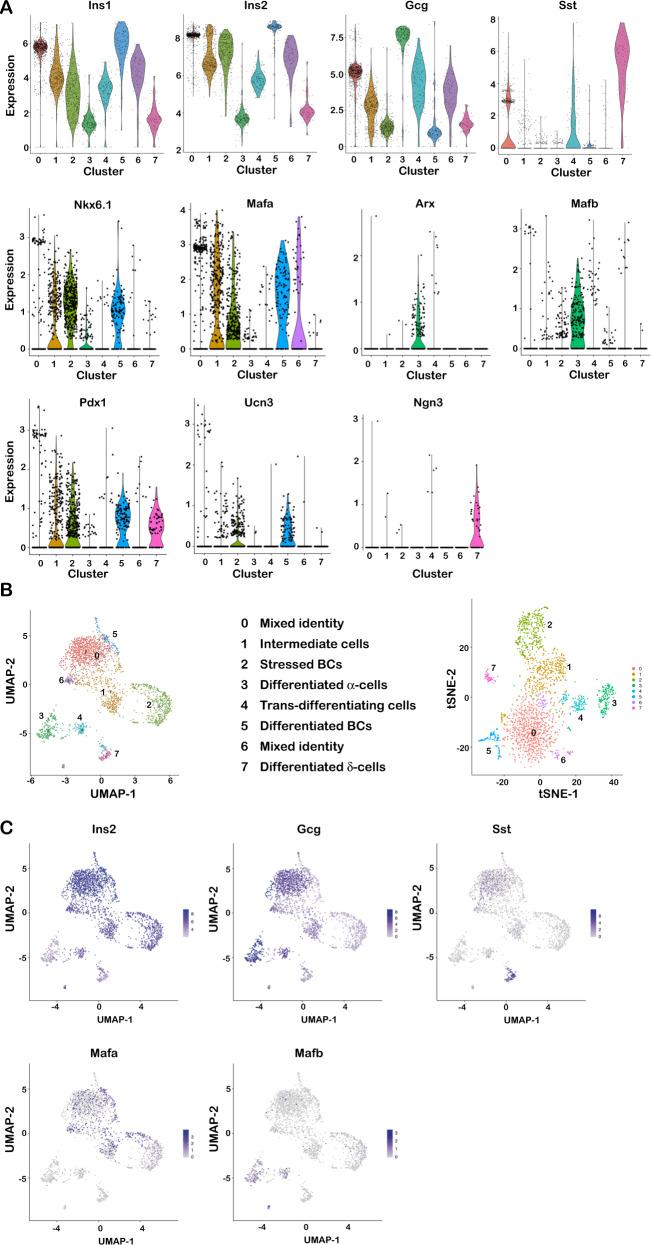
Sc-RNA-seq of week 1 mutant islets. **A** Violin plots of expression of the indicated genes in each cluster. **B** UMAP (left) and tSNE (right) representations of the cell populations. **C** UMAPs illustrating the expression of the genes indicated in each panel.

We analysed 2295 cells 1 week after Taf4 inactivation. Clusters 2 and 5 were the most differentiated beta cell populations with high *Ins2* and *Ucn3* and lowest levels of *Gcg* and *Sst* (Fig. 5A). Cluster 2 however was marked by ER stress (Supplementary Fig. 6D). Cluster 0 showed higher *Gcg* and *Sst*, although lower than in differentiated alpha and delta cell populations, clusters 3 and 7, respectively, that segregated from the beta cell populations in the UMAP and tSNE representations (Fig. 5B, C and Supplementary 6B). The small cluster 6 also showed characteristics of a mixed identity population expressing *Ins1*/*2* and intermediate levels of *Gcg*. Taf4 inactivation therefore led to several populations with mixed identity expressing *Ins2*, but also *Gcg* or *Sst* and sometimes all three.

Cluster 1 were intermediate cells with lower *Ins2, Ucn3* and higher *Gcg* than the more differentiated clusters 2 and 5. Cluster 4 expressed intermediate levels of *Ins2*, *Gcg* and *Sst* and segregated between the beta, alpha and delta cell populations on the UMAP. Cluster 4 cells lost expression of beta cell-markers *Pdx1*, *Mafa* and *Nkx6.1* and appeared to be trans-differentiating intermediates between beta and alpha cell-like identities (Fig. 5C and Supplementary 6B). Ontology analyses (Supplementary Fig. 6C and Dataset 2) showed cluster 5 was marked by differentiated beta cell genes, cluster 2 showed translational activity, ER stress associated with high *Ddit3* and *Herpud1* and high oxidative phosphorylation (Supplementary Fig. 6D). In contrast, signatures for transcription regulation, chromatin modification and RNA metabolism were hallmarks of clusters 1 and 4 consistent with the idea that these cells were undergoing transcriptional reprogramming.

**Fig. 6:**
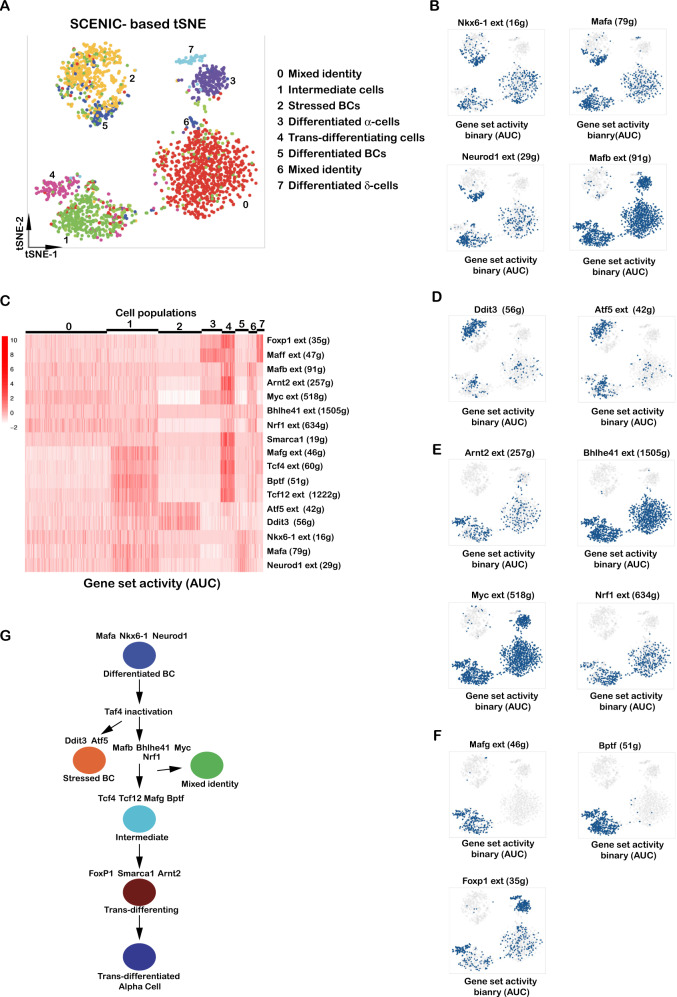
Transcription factors regulating cell state in week 1 mutant islets. **A** SCENIC-based tSNE representation colouring cells based on the binary activities of the transcription factor regulons. **B**, **D**–**E** Binary activities of the transcription factor regulons in the different cell populations. **C** Heatmap of regulon activities in the different cell populations were quantified using AUCell. **G** A flow-chart of the progression of cell state from differentiated beta cells to trans-differentiated alpha cells. The SCENIC regulons marking the transitions of each state are indicated.

We used SCENIC [42] to identify transcription regulatory networks active in the different cell populations. The SCENIC-based tSNE (Fig. 6A) that groups cells based on their regulon activities differed from the expression based tSNE (Fig. 5B), with the beta-cell clusters 2 and 5 grouped together, clusters 1 and 4 grouped closely, while cluster 0 grouped close to alpha cells (Fig. 6A). Consistent with the idea that cluster 5 were differentiated beta cells, they showed high activity of the Mafa, Nkx6-1 and Neurod1 regulons (Fig. 6B, C) that were active in cell sub-populations of clusters 0 and 1, but absent from alpha and delta cells. Cluster 2 was marked by activity of Ddit3 and Atf5, that promotes beta-cell survival under stress [43], consistent with their elevated stress response (Fig. 6C, D). In contrast, Mafb activity was seen in alpha cells and in clusters 0, 1 and 4 confirming they represented abnormal populations with mixed identities, some showing both Mafa and Mafb activities (Fig. 6B, C).

Strikingly, clusters 0, 1 and 4 showed activities of the Arnt2, Bhlhe41, Myc and Nrf1 regulons in line with enhanced binding predicted from ATAC-seq footprinting (Fig. 6C and E). Clusters 1 and 4 were specifically marked by activity of the small Maf inhibitor Mafg, Tcf4, Tcf12 and the Bptf subunit of the NuRF complex coherent with their chromatin remodelling ontology signatures (Fig. 6C and E). The Foxp1 and Maff regulons showed highest activity in cluster 4 and in differentiated alpha and/or delta cells (Fig. 6C and F). Mafb, Myc, Arnt2, Mafg, Tcf4, Tcf12, Bptf and Foxp1 regulons showed highest combinatorial activity in cluster 4. Moreover, specific activity of Smarca1, the catalytic NuRF complex subunit, in cluster 4 together with Bptf activity suggested a critical role of the NuRF complex in trans-differentiation (Fig. 6C). These results were consistent with a major transcriptional reprogramming and chromatin remodelling associated with trans-differentiation that did not appear to involve transition via a precursor state as low *Ngn3* expression was seen in the delta cell population, but not in clusters 1 and 4 (Fig. 5A). As a result, by 5 weeks two alpha cell populations were observed potentially corresponding to the normal and trans-differentiated populations.

At 5 weeks, no populations with mixed or obviously trans-differentiating characteristics were observed suggesting that trans-differentiation took place immediately after Taf4 loss. SCENIC analyses on 2500 randomly selected cells from the *Taf4*^+/+^/W5 aggregate (Supplementary Fig. 7A) showed that *Taf4*^+/+^ beta cells were marked by Mafa and the glucose-sensing transcription factor Mlxipl, highest in populations 0 and 6 (Supplementary Fig. 7B). Mafa activity was also strong in the *Taf4*^−/−^ beta cell populations with cluster 11 displaying high activity of both Mafa and Mlxipl. Mafb was active in the alpha cell populations, but contrary to week 1 no overlap of Mafa and Mafb activities was seen. Several *Taf4*^+/+^ beta cell populations characterized by stress response as described above did however show activity of the Mafg, Yy1, Atf5, Atf3, and Bhlhe41 regulons together with those of Jun and Fosb (AP1). Similarly, the stressed *Taf4*^−/−^ alpha cells also showed elevated AP1 and Atf3 activity. *Taf4*^−/−^ beta cells also showed elevated activity of chromatin remodelling factor Chd2. In contrast, activity of all of these regulons was much lower or absent in *Taf4*^+/+^ beta cells.

Trans-differentiation of a sub-population of beta cells into Gcg-expressing apha-like cells was supported by Ins and Gcg co-immunostaining (Supplementary Fig. 8A). In *Taf4*^+/+^, Gcg staining was limited to alpha cells surrounding the islet. By 3 weeks after Taf4 inactivation, Gcg-expressing cells were observed both around and amongst the Ins-expressing beta cells, a phenomenon seen later stages and analogous to loss of Pdx1 that promoted beta cell trans-differentiation into Gcg-expressing cells [44]. Such cells were not co-stained with Ins, but labelled uniquely by Gcg.

To confirm beta cell trans-differentiation, we crossed the *Ins1*::CreER^T2^::*Taf4*^lox/lox^ animals with mice bearing a Lox-Stop-Lox cassette driving a GFP reporter in the *Rosa26* locus. Tam administration deleted the Lox-Stop-Lox cassette and activated GFP expression in the beta-cell compartment (Fig. S8B). At 12 weeks after Tam injection, staining with Gcg identified bone-fide alpha cells at the periphery of the islets that were negative for GFP, whereas within the islets, most Gcg-expressing cells were GFP positive indicating they trans-differentiated from beta cells.

## Discussion

### Taf4 is essential for normal beta-cell function

Taf4 inactivation in adult murine beta cells led to increased glycaemia due to defective insulin signalling and secretion, a consequence of an immediate and major impact on gene expression. Immunostaining showed a rapid and efficient loss of Taf4 in beta cells by 1 week after Tam injection. At 34 weeks, Taf4-expressing beta cells were again detected, but their proportion did not increase by 55 weeks. While it is possible that Taf4-expressing beta cells detected at later times arose from rare non-recombined cells, no Taf4 expressing beta cells were observed upon staining of multiple islets at regular intervals from 3–24 weeks, whereas they were readily observed at 34 and 55 weeks. Furthermore, the number of Taf4-expressing beta cells did not increase between 34 and 55 weeks arguing against proliferation of non-recombined beta cells. Thus, while we cannot definitively exclude the idea that Taf4-expressing cells arose from non-recombined beta cells, an alternative possibility is that they arose from trans-differentiation of alpha cells or other islet populations into beta cells as has been previously described, for example under conditions of reduced beta cell-mass [2, 5, 6, 12, 45]. Notwithstanding the mechanisms involved, despite chronically high glycaemia, the beta-cell population was only partially and slowly replaced by newly generated Taf4-expressing beta cells. Moreover, the Taf4 expressing beta cells seen at later times cannot account for the partially restored glucose and insulin levels seen at 10 weeks, but they may account from the longer-term viability of the animals

### A model for beta-cell trans-differentiation

While there have been several scRNA-seq data sets generated from human adult islet cells [46], studies on adult murine islets have been more limited [46, 47]. We distinguished several beta cell populations in *Taf4*^+/+^ and *Taf4*^−/−^ animals with distinct gene expression signatures. Cells with high insulin expression were distinguished from populations with low insulin, but high expression of insulin signalling genes and from a population with high expression of ER stress genes. This is consistent with previous data [46, 47] and the idea that high insulin levels induce ER stress prompting cells to transit into a high ER stress/low insulin recovery state. Taf4 inactivation led to a stronger ER stress in a subset of both beta and alpha cells. Wild-type delta cells also displayed high ER stress that was increased upon Taf4 inactivation. This increased cell non-autonomous ER stress after Taf4 inactivation suggested defective communication between the *Taf4*^−/−^ beta cells and their alpha and delta cell neighbours.

Bulk ATAC-seq and RNA-seq showed that Taf4 inactivation had a potent impact on chromatin accessibility and gene expression with diminished binding of beta cell identify factors. In contrast, increased binding of several transcription factors was predicted from ATAC-seq and several of the corresponding regulons were activated in the abnormal *Taf4*^−/−^ cell populations. At week 1, cells with mixed identities displayed regulon activity of beta-cell factors and the alpha cell determinant Mafb, but did not express *Arx* and therefore displayed an incomplete switch in identity in line with the intermediate levels of *Gcg*. Previous studies on normal mouse or human pancreas by immune-staining or scRNA-seq detected only rare cells simultaneously expressing two of the islet hormones [9, 10, 47–50]. Such cells have however been detected in pathological situations like type 1 and type 2 diabetes and in mice following loss of Arx and Dnmt1 [9]. The mixed cell identity was therefore a consequence of Taf4 inactivation and activation of novel regulons.

Our data pointed to a process of stress-induced beta-cell trans-differentiation. Cluster 4 cells displayed strongly decreased expression and activity of beta-cell factors, but showed Mafb activity, increased *Arx* and higher *Gcg* and *Sst* expression. Furthermore, they were marked by Foxp1 consistent with the essential role of Foxp factors in alpha cells [51] and by activity of Maff, Mafg and the NuRF complex. Inhibition of (s)Maf proteins in beta cells led to increased Mafa activity and beta-cell function correlating with the increased Mafg/Maff activity and loss of beta-cell identity in the trans-differentiating cells found here [52, 53].

We propose a model for beta-cell trans-differentiation involving a progressive loss of beta-cell identity associated with gain of activity for Myc, Mafg, Arnt2, Bhlhe41, Tcf4, Tcf12, Nrf1 and Mafb (Fig. 6G). A population of cells further undergoes chromatin remodelling and transcriptional reprogramming through the combinatorial action of these factors and subsequent acquisition of Foxp1, Maff and Smarca1 activity that promotes trans-differentiation into *Gcg*-expressing alpha-like cells. Trans-differentiation was further attested by the presence of Gcg-expressing cells intermixed amongst beta cells, and by lineage tracing that confirmed that most Gcg-expressing cells within the islets were indeed derived from beta cells.

Mechanisms of trans-differentiation of alpha to beta-like cells have been documented [4, 8, 9, 46] and examples of beta to alpha-like cell trans-differentiation often involve genetically induced loss of beta cell determinants [11, 14, 44]. In this study, beta cell trans-differentiation also involved loss of beta cell-determinants perhaps triggered by the transient stress and inflammatory response seen 1 week after Taf4 inactivation. This inflammatory/stress environment may be responsible for activation of Myc and previously implicated in beta cell de-differentiation [54–56].

In previous studies of trans-differentiation, the beta-cell populations actively undergoing trans-differentiation and the associated transcriptional regulatory programs involved are poorly characterized. Using an un-supervised bioinformatics approach, we defined how novel combinations of known critical beta cell and alpha cell determinants act in combination with additional transcription and chromatin remodelling factors to promote beta-cell trans-differentiation. While different cues may underlie initiation of beta-cell trans-differentiation in pathological situations or following Taf4 inactivation, further studies will determine whether they share common aspects of the transcriptional reprogramming described here.

## Supplementary information

Legends to Supplemental Figures

Supplemental Figure 1

Supplemental Figure 2

Supplemental Figure 3

Supplemental Figure 4

Supplemental Figure 5

Supplemental Figure 6

Supplemental Figure 7

Supplemental Figure 8

Supplemental Dataset 1

Supplemental Dataset 2

## Data Availability

Source data for this paper are available from the authors upon reasonable request. All sequencing data reported here have been submitted to the GEO database under accession number GSE151366.
