# Supplementary material for: Single cell transcriptomics reveal trans-differentiation of pancreatic beta cells following inactivation of the TFIID subunit Taf4
Source: Cell Death Dis. 2021 Aug 12;12(8):790. doi: 10.1038/s41419-021-04067-y (PMC8361202; doi:10.1038/s41419-021-04067-y)
Supplement: Supplementary file 1 — Legends to Supplemental Figures [file 41419_2021_4067_MOESM1_ESM.docx]

**Legends to Supplementary Figures.**

**Supplementary Figure 1. A.** Enlarged view of islets from control or Taf4-mutant animals stained for Ins and Taf4. White * show Taf4 expressing nuclei (or groups of Taf4 expressing nuclei) while yellow * indicate nuclei where Taf4 is absent. **B**. Staining of frozen islets for Gcg and Taf4. Peripheral Taf4 and Gcg-expressing alpha cells are indicated by *.

**Supplementary Figure 2.** Changes in gene expression upon Taf4 inactivation. **A.** Venn diagrams summarizing genes up and down-regulated 1, 3 and 5 weeks after Tam injection. **B**. Volcano plots showing changes in gene expression after 1, 3 and 5 weeks. **C.** Summary of RNA-seq data on the expression of selected up and down-regulated genes critical for beta cell function, identity, cell-cell contact, stress response and alpha cell and delta cell markers. **D**. Heatmap showing the expression of the 100 genes most up and down-regulated 3 weeks after Taf4 inactivation. Right hand panel shows ontology analyses of the de-regulated genes.

**Supplementary Figure 3.** Changes in chromatin accessibility in dissociated islets 1 and 5 weeks after Taf4 inactivation measures by ATAC-seq. **A-B**. Scatterplots showing Log10 changes in ATAC-seq peaks either globally (left panels) or at the proximal promoter (-1000/+100 base pairs relative the transcription start site, TSS, right panel) at 1 week (A) or 5 weeks (B) following TAF4 inactivation. The number of peaks enriched in each condition are indicated. **C-D**. In silico footprinting using Tobias. Scatter plots of differential footprinting depth of transcription factor motifs in differentially accessible regions between WT and week 1 (C) and week 5 (D). Motifs with more footprint depth in WT are labelled in pink and in Taf4 mutants in light blue.

**Supplementary Figure 4.** Heatmap of differentially expressed genes in the cell clusters from scRNA-seq of WT islets.

**Supplementary Figure 5.** Heatmap of differentially expressed genes in the cell clusters from the WT/week 5 aggregate data.

**Supplementary Figure 6 A**. UMAP representations of the aggregate data illustrating the expression of the genes indicated in each panel. **B** tSNE representations of the week 1 cell populations illustrating the expression of the genes indicated in each panel. **C.** Bubble plots showing ontology (BP-FAT) of genes differentially expressed in the indicated clusters. **D**. UMAP representations week 1 cell populations illustrating the expression of the genes indicated in each panel.

**Supplementary Figure 7.** SCENIC analyses of WT and 5 week mutant islets populations. **A.** SCENIC based tSNE of 2500 cells from the WT/W5 aggregate. The identities of the cell populations are indicated. **B.** Heatmap representation of regulon activities in the different cell populations were quantified using AUCell.

**Supplementary Figure 8. A.** Immunostaining of Langerhans islets from mice with the indicated genotypes for Insulin, Gcg or DAPI as indicated. The number of weeks after Tam injection are indicated. The Insulin-Gcg merge is shown to illustrate the mutually exclusive nature of the labelling. **B.** Immunostaining of Langerhans islets from *Taf4^b-/-^* mice crossed with Rosa 26 Lox-Stop-Lox GFP mice 12 weeks after Tam injection with antibody for Gcg, DAPI and GFP fluorescence as indicated. Representative GFP-negative-Gcg-expressing alpha cells at the islet periphery are indicated along with representative GFP-positive-Gcg-expressing cells within the islet indicated by white arrows. Scale bar = 100 μM

**Supplementary Dataset 1.** Summary of RNA-seq 1 3 and 5 weeks after Taf4 inactivation. Each spreadsheet shows the genes up (UR) and down-regulated (DR) along with the corresponding ontology analyses.

**Supplementary Dataset 2.**  Summary of genes differentially regulated in the cell populations 1 week after Taf4 inactivation along with their ontologies.
