# Supplementary figures and images for: Single cell transcriptomics reveal trans-differentiation of pancreatic beta cells following inactivation of the TFIID subunit Taf4
Source: Cell Death Dis. 2021 Aug 12;12(8):790. doi: 10.1038/s41419-021-04067-y (PMC8361202; doi:10.1038/s41419-021-04067-y)

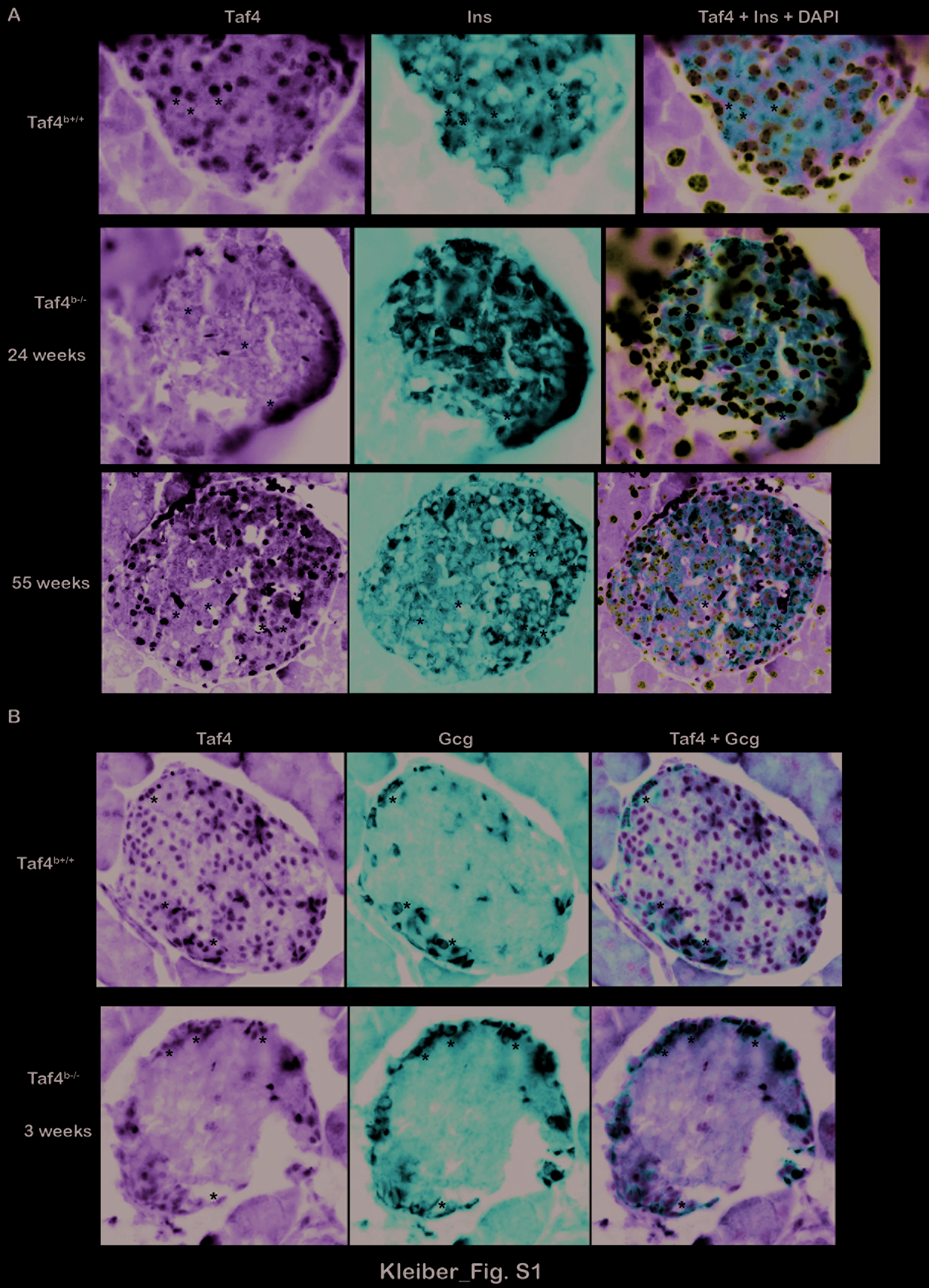

Supplement: Supplementary file 2 — Supplemental Figure 1 [file 41419_2021_4067_MOESM2_ESM.tif]

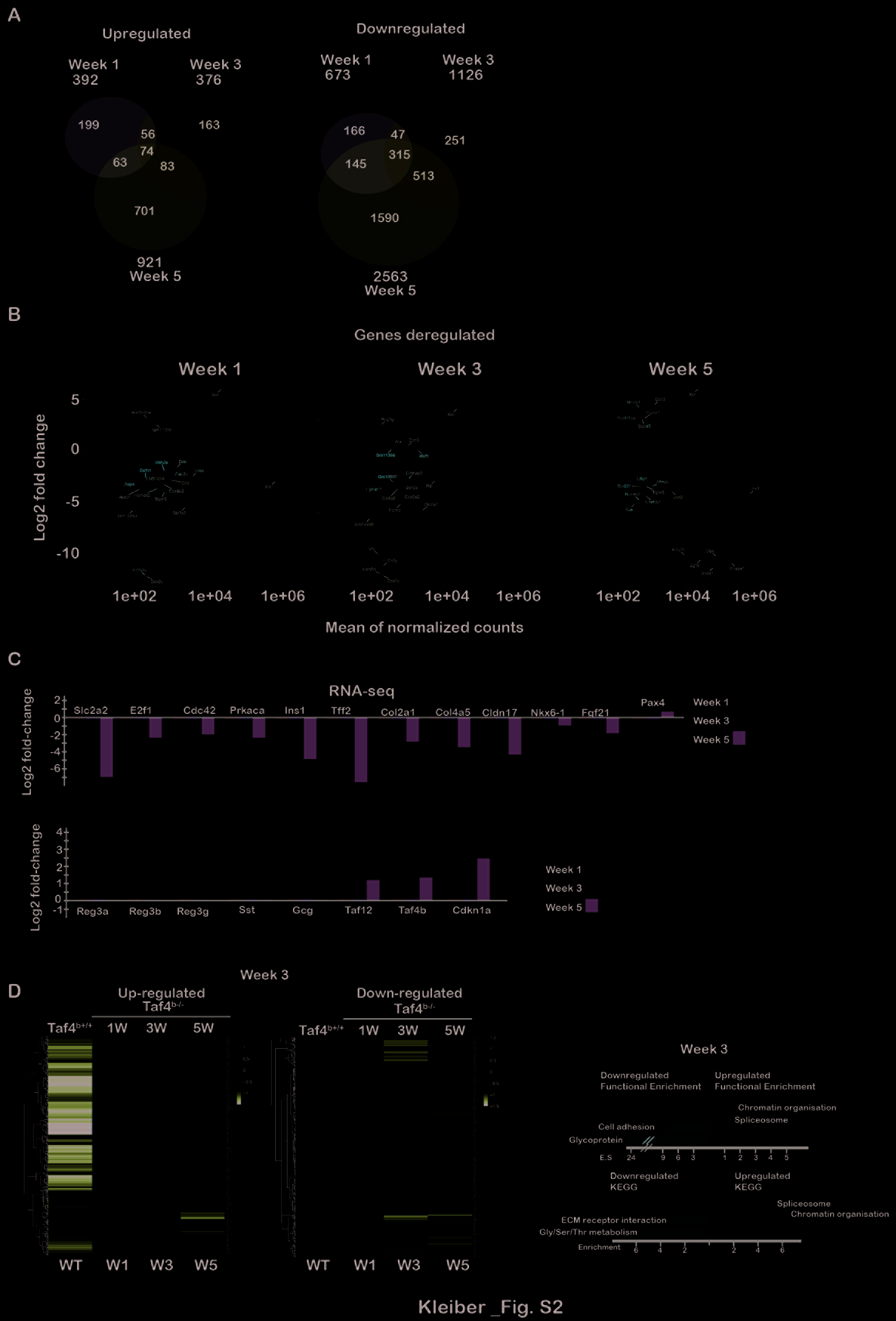

Supplement: Supplementary file 3 — Supplemental Figure 2 [file 41419_2021_4067_MOESM3_ESM.tif]

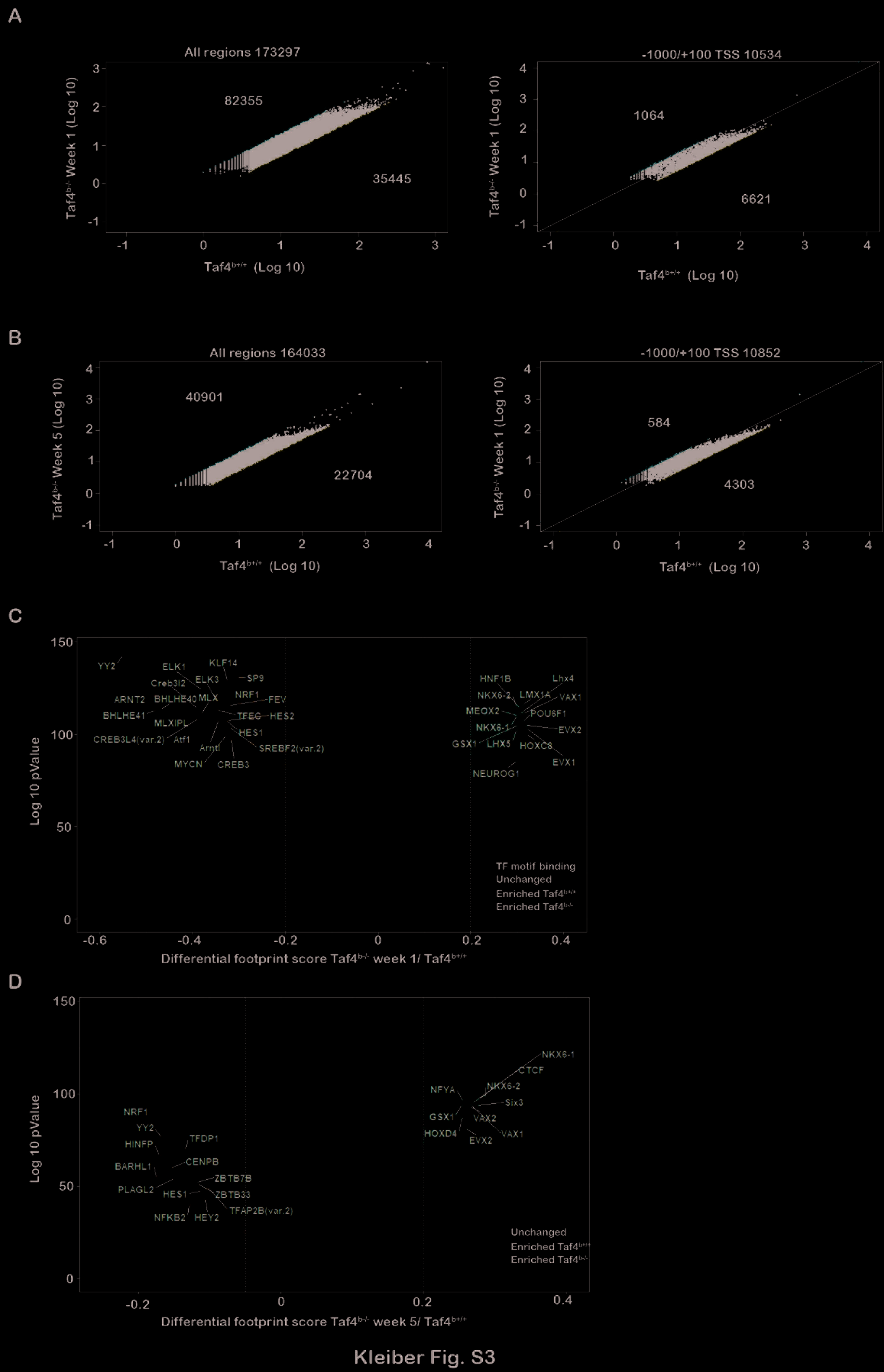

Supplement: Supplementary file 4 — Supplemental Figure 3 [file 41419_2021_4067_MOESM4_ESM.tif]

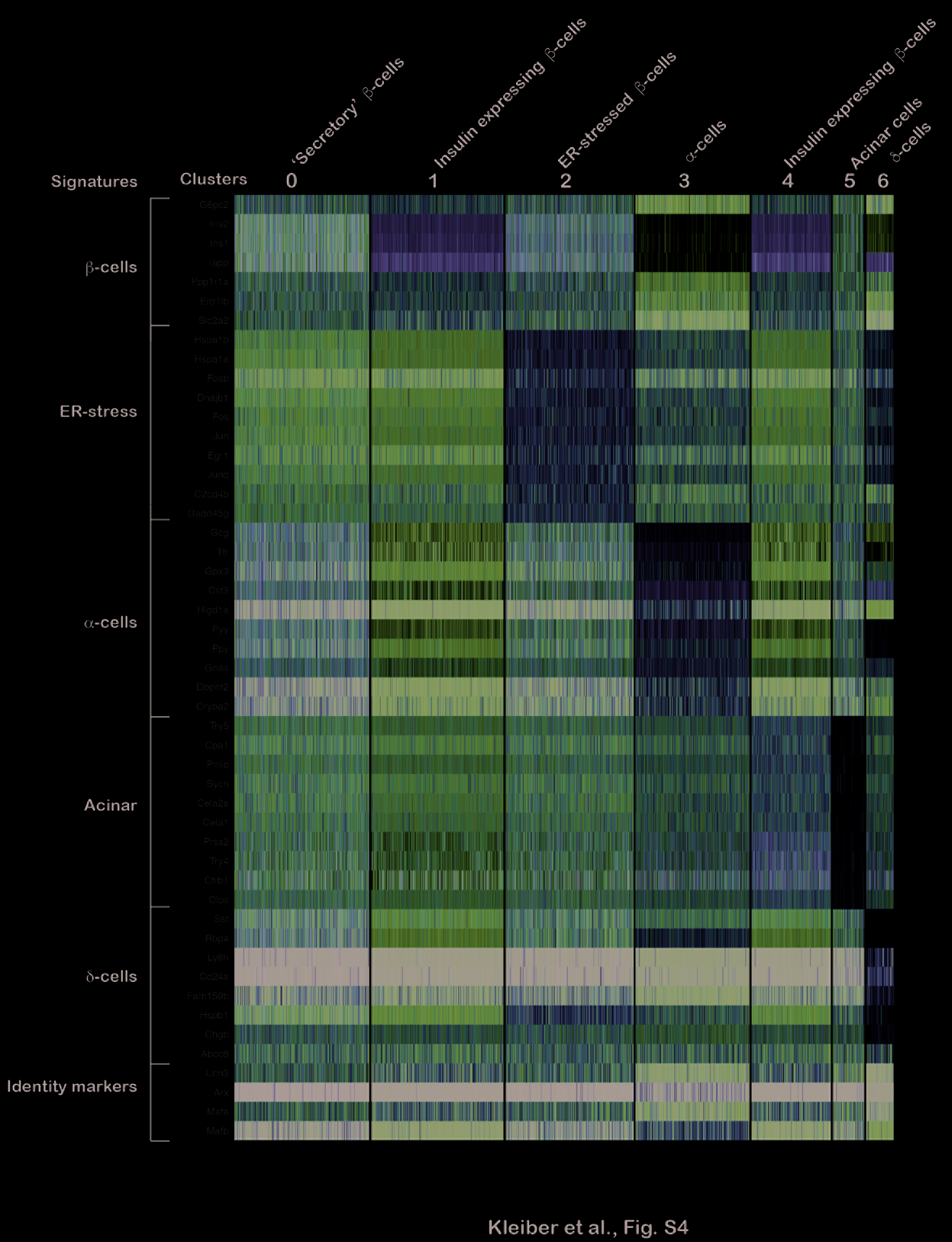

Supplement: Supplementary file 5 — Supplemental Figure 4 [file 41419_2021_4067_MOESM5_ESM.tif]

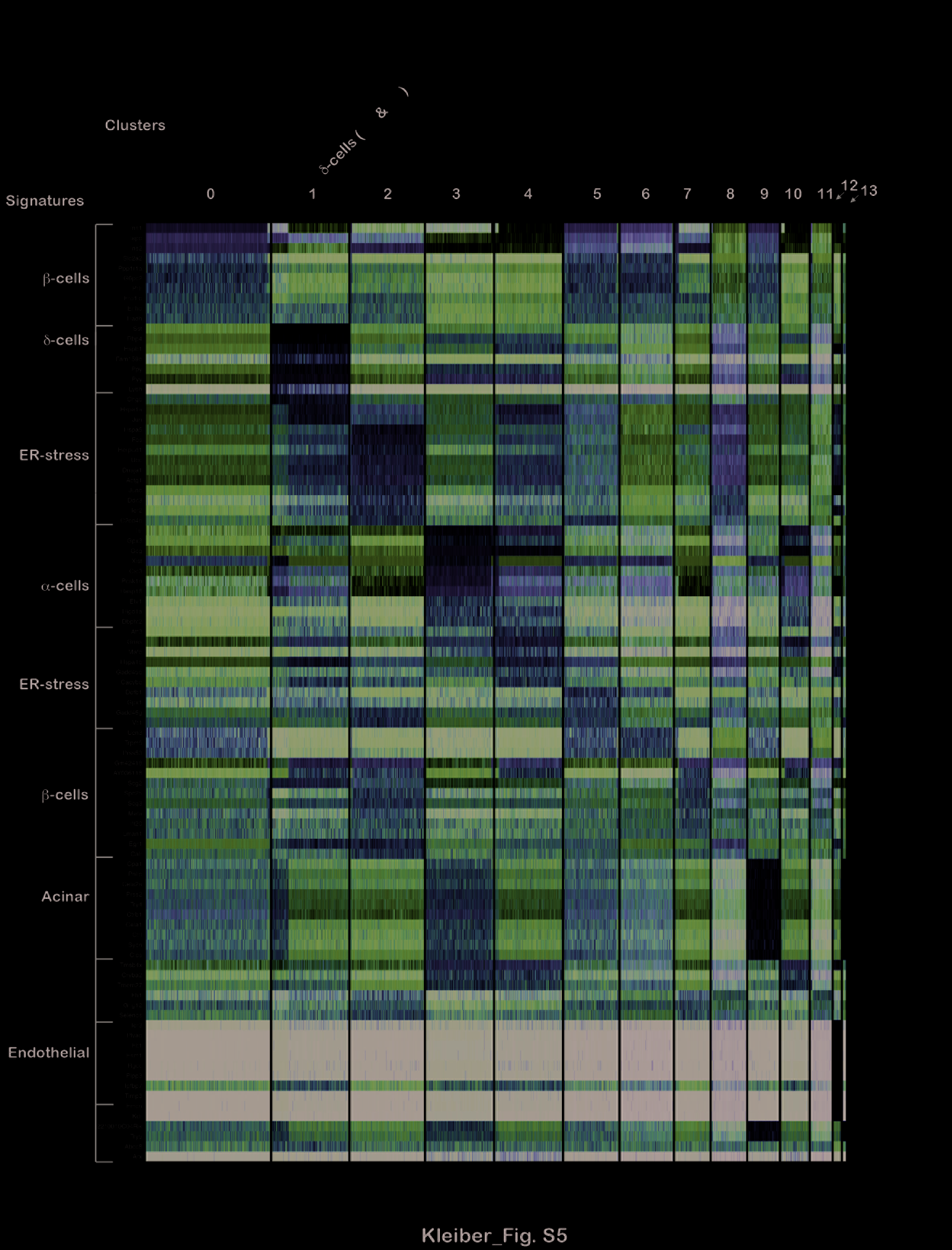

Supplement: Supplementary file 6 — Supplemental Figure 5 [file 41419_2021_4067_MOESM6_ESM.tif]

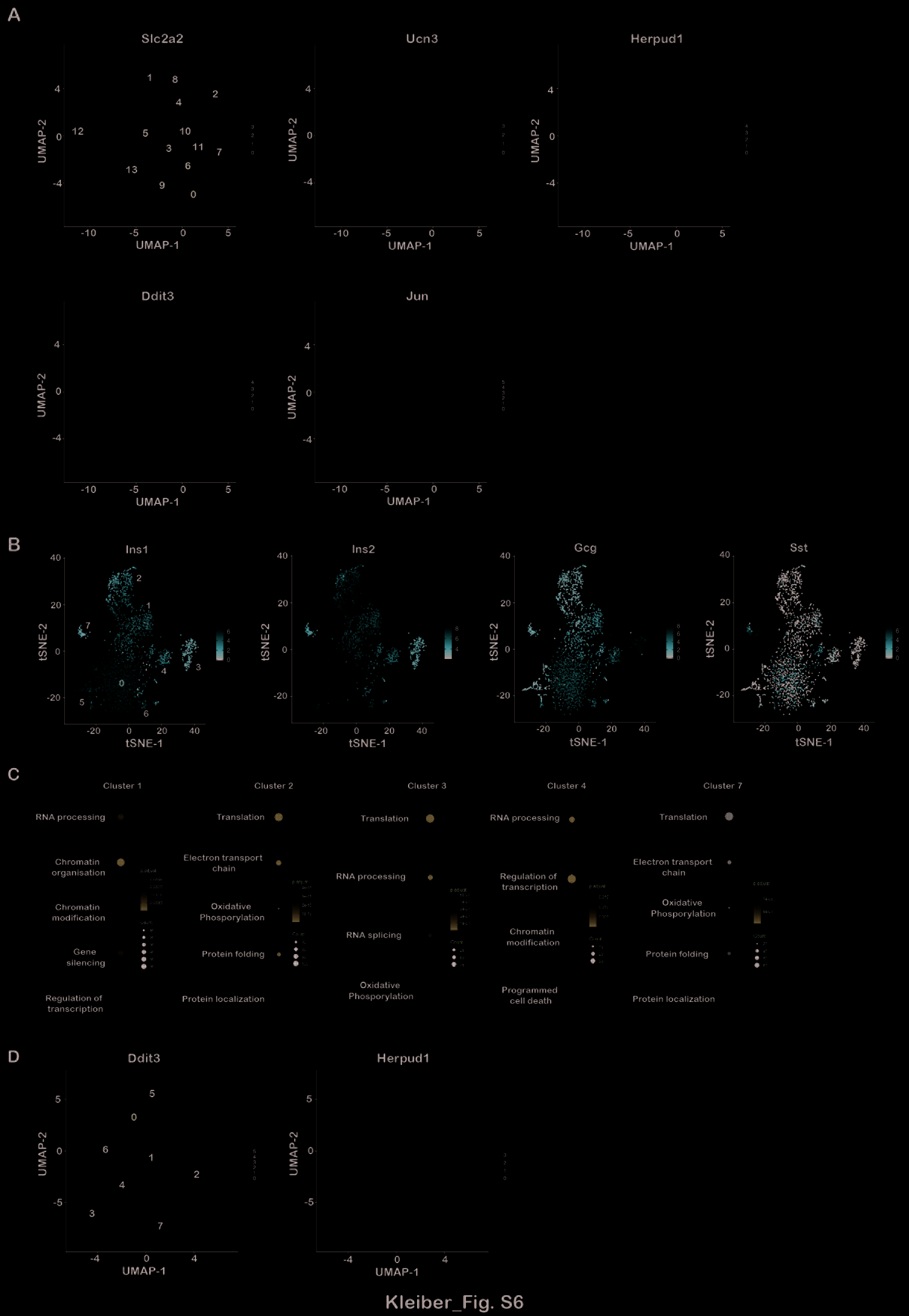

Supplement: Supplementary file 7 — Supplemental Figure 6 [file 41419_2021_4067_MOESM7_ESM.tif]

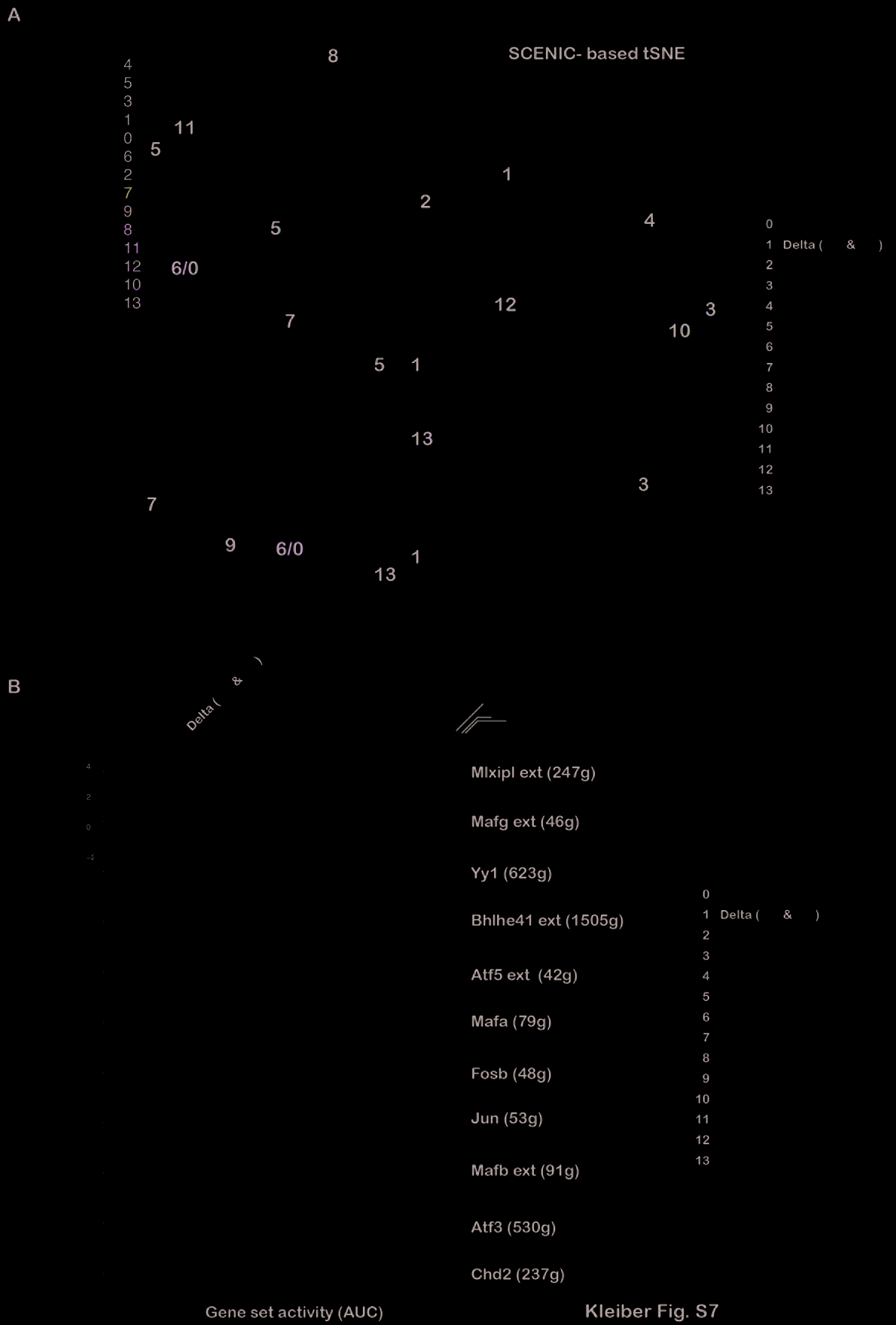

Supplement: Supplementary file 8 — Supplemental Figure 7 [file 41419_2021_4067_MOESM8_ESM.tif]

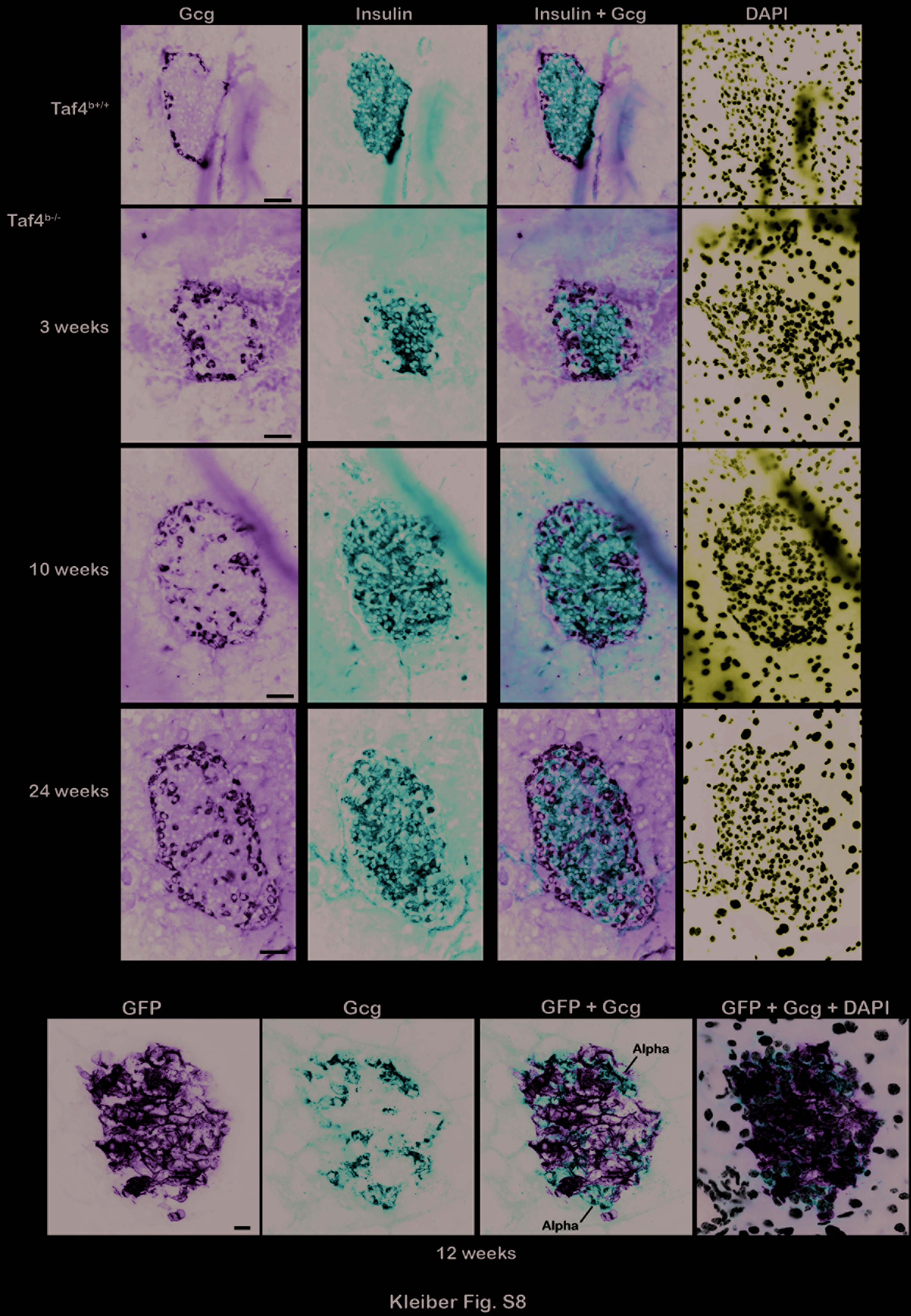

Supplement: Supplementary file 9 — Supplemental Figure 8 [file 41419_2021_4067_MOESM9_ESM.tif]
